# Role of serum cytokeratin 19 fragment (Cyfra 21.1) as a prognostic biomarker in patients with differentiated thyroid cancer

**DOI:** 10.1038/s41598-017-07915-0

**Published:** 2017-08-04

**Authors:** L. Giovanella, M. Imperiali, P. Trimboli

**Affiliations:** 1 Oncology Institute of Southern Switzerland, Department of Nuclear Medicine and Thyroid Centre, 6500 Bellinzona, Switzerland; 2 Ente Ospedaliero Cantonale, Department of Clinical Chemistry and Laboratory Medicine, 6500 Bellinzona, Switzerland

## Abstract

Differentiated thyroid cancers (DTC) account for up to 85% of thyroid cancers and generally display an excellent prognosis. However, in a minority of cases, DTC progress toward less differentiated phenotypes leading to distant metastases and even disease-related deaths. Circulating biomarkers are warranted to complement the gold standard DTC marker thyroglobulin (Tg) in identifying and monitoring such cases. We measured serum Tg and Cyfra 21.1 6 to 12 months after primary treatment in 473 DTC patients. A complete response of Tg was related to an excellent outcome in all cases. Among patients with incomplete Tg response Cyfra 21.1 levels <2.07 ng/mL were associated to favorable outcome while higher levels greatly increased the risk of disease related recurrences and deaths. Both markers retained independent prognostic values in multivariate analysis. In conclusion, Cyfra 21.1 is a tool available to independently predict survival of DTC patients not achieving excellent response after primary treatment.

## Introduction

Tumours derived from thyroid epithelial cells are the most frequent endocrine neoplasms and include a large spectrum of overlapping lesions that differ in tumour biology and range from slow-growing differentiated thyroid carcinoma (DTC) to the poorly differentiated thyroid carcinoma and the rapidly fatal anaplastic thyroid carcinoma, respectively^1^. DTC account for 80–85% of thyroid cancers; even if their prognosis is generally excellent approximately one-third of patients recur over time, with distant metastasis present in about 20% of cases. Of note, about half of patients with distant metastases die within 5 years^2^. In these cases the ability to concentrate radioiodine and to synthesize and secrete thyroglobulin (Tg) is frequently reduced (i.e. tumor cell de-differentiation) leading to a poor responsiveness to conventional therapy and difficulty cancer monitoring by serum Tg measurement^3, 4^. Therefore, new circulating biomarkers are warranted to help identify and monitor patients carrying dedifferentiating tumours^5, 6^. The cytokeratin 19 (CK19) is an acidic protein of 40 kDa that is part of the cytoskeleton of epithelial cells. Tissue CK19 is highly expressed in DTC, mainly those with papillary histotype (PTC)^7, 8^ and CK19 immunostaining may be useful in thyroid pathology as a supplement to classical histological and/or cytological analysis^9, 10^. Another clinical application is the detection of soluble protein fragments derived from CK19 (Cyfra 21.1) in the circulation of cancer patients. These fragments, released by carcinoma cells, are employed as a diagnostic or prognostic marker in lung, breast, head and neck, gynecological and bladder cancers^11–13^. More recently, serum CK19 fragments (Cyfra 21.1) emerged as potentially useful circulating marker in dedifferentiating thyroid cancer despite low or absent CK19 immunostaining in corresponding tumour tissues carcinomas^7, 14–16^. Previous studies in human lung and liver cancer cell lines well explain this apparent paradox as high Cyfra 21.1 levels in culture supernatants were obtained only in cultured tumours expressing caspase-3 (an enzyme involved in apoptosis phenomena)^17, 18^. As a consequence, only tumors with a high apoptosis rate and fast processing of CK19 molecules release CK19-soluble fragments in the bloodstream with coexisting negative tissue CK19 immunostaining and high serum Cyfra 21.1 level^19^. Accordingly, both caspase-3 enzyme activity^5^ and increased serum Cyfra 21.1 levels^20, 21^ were found in DTC with aggressive behaviour. Then the present study was undertaken in a large population of DTC patients to evaluate the role of serum Cyfra 21.1 measurement in predicting biological DTC behaviour and clinical outcome of patients over time.

## Results

### Patient characteristics and general results

Among 1127 retrieved patients a series of 536 DTC patients fulfilling the criteria was enrolled. After exclusion of 63 TgAb-positive patients a final series of 473 patients was selected for the present study. Demographic, clinical and pathologic characteristics, OS and DFS of these patients are summarized in Table 1. Eight patients (1.6%) patients died for disease-related causes while remaining 465 ones (98.4%) were alive at the end of the follow-up. Among them 62 (13%) patients had structural (37 loco-regional and 25 distant sites) recurrences (sREC) and other 376 (80%) were disease-free (NED, no evidence of disease). The remaining 35 (7%) patients showed biochemical recurrences and were excluded from final statistical analysis. The frequency of cancer-related deaths and sREC increased in high-risk DTC [6 (13%) and 19/40 (47%)] compared to intermediate-risk [2 (1%) and 32/138 (23%)] and low-risk [0 and 11/295 (4%)] ones (p < 0.0001). Patients with sREC were older than NED ones (with more patients > 55 years old), had more follicular than papillary cancers and carried larger primary cancers at initial postsurgical work-up (Table 2). Among 62 patients having sREC loco-regional recurrences and distant metastases were detected in 34 (55%) and 28 (45%) cases, respectively. Distant metastases involved lungs (12 patients), bone 7 patients), soft tissues (2 patients), liver (2 patients) and multiple organs (5 patients). Among the latter, distant metastases involved lung and soft tissue (1 patient), lung and bone (2 patients), bone and liver (1 patient), and lung, soft tissue, liver, kidney and and bone (1 patient), respectively. Eigth of 28 patients (29%) with distant metastases died for disease-related causes with a median OS of 11 (range 5–36) months.

**Table 1:** Patients’ clinico-pathological characteristics and survival data.

**Gender**	
Females	347 (73%)
Males	126 (27%)
**Age (** ***years*** **)**	
Age at cancer diagnosis	49 (18–88)
**Histology**	
PTC	398 (84%)
FTC	75 (16%)
**Risk categories**	
Low	295 (62%)
Intermediate	138 (30%)
High	40 (8%)
**Radioiodine ablation**	
Administered activity (GBq)	3 (1.1–7.4)
**Overall Survival** (*months*)	
Overall series (n = 473)	36 (6–140)
Died patients (n = 8)	11 (5–36)
Alive patients (n = 465)	45 (25–140)
**Disease Free Survival (** ***months*** **)**	
Overall series (n = 473)	36 (6–140)
NED (n = 376)	45 (29–140)
bREC (n = 35)	35 (12–140)
sREC (n = 62)	23 (5–128)

**Table 2:** Comparison between NED and sREC patients’ characteristics.

	**NED** (n = 376)	**sREC** (n = 62)	p
**Age**			
Age at diagnosis (yrs)	49, 21 to 88	65, 26 to 79	0.03
Age > 45 yrs	259, 68.8%	44, 71.0%	0.49
Age > 55 yrs	148, 39.3%	35, 56.4%	0.004
**Histotype**			
PTC	336, 89.4%	52, 83.3.%	0.009
FTC	40, 10.6%	10, 16.7%	
**ATA risk stratification**			
Low	225, 59.8%	15, 24.2%	0.0003
Intermediate	133, 35.3%	31, 50%	
High	18, 4.9%%	16, 25.8%	
**pTNM**			
pT1	219, 58.2%	10, 16.1%	0.0001
pT2	87, 23.2%	23, 37.0%	
pT3	67, 17.8%	25, 40.4%	
pT4	3, 0.8%	4, 6.4%	
pN1	89, 23.6%	27, 43.5.%	0.07
pM1	0	6, 9.7%	0.005
**Stage**			
Stage I	184, 48.9%	6, 9.6%	<0.0001
Stage II	101, 26.8%	27, 43.6%	
Sage III	87, 23.3.%	21, 33.9%	
Stage IV	4, 1.0%	8, 12.9%	

### Serum thyroglobulin and Cyfra 21.1 in detecting cancer relapse and predicting outcome over time

**Figure 1:**
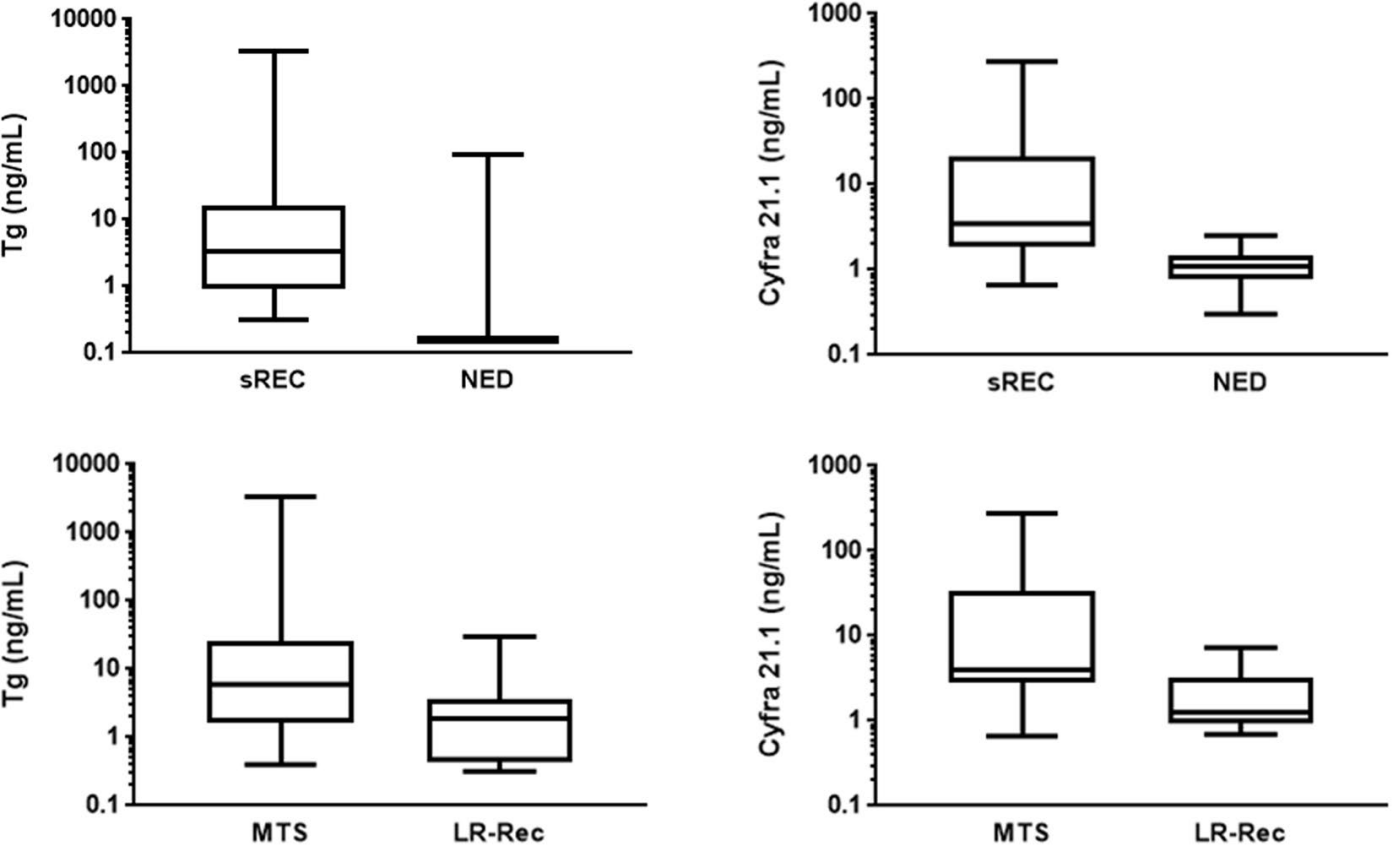
Box-plot analysis of serum Tg and Cyfra 21.1 distribution in patients with structural recurrence (sREC) versus NED and loco-regional recurrences (LR-Rec) versus distant metastases of DTC (MTS).

**Figure 2:**
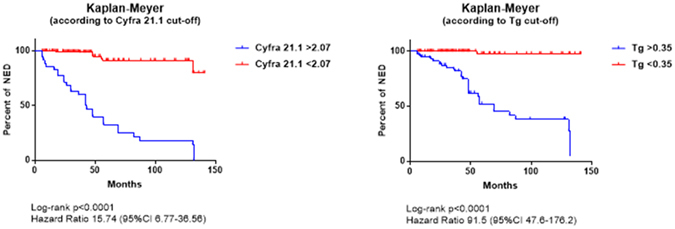
Kaplan-Meier curves of event-free survival according to Tg and Cyfra 21.1 value below or above the most accurate cut-offs selected by ROC curve analysis in our series

**Figure 3:**
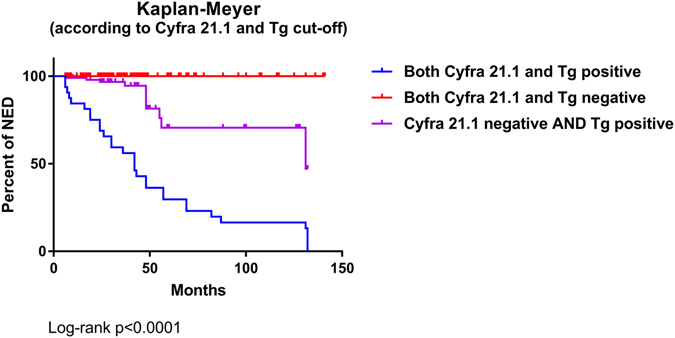
Kaplan-Meier curves of event-free survival according to combined Tg and Cyfra 21.1 values Note: due to the low number of patients and events the curve of Cyfra positive/Tg negative patients was omitted.

Serum Tg and Cyfra 21.1 levels were measured at first follow-up visit and compared with patients’ outcome over time. At ROC curve analysis the most accurate Tg and Cyfra 21.1 cut-off values to discriminate NED and sREC patients were 0.35 ng/mL (Area Under the Curve 0.950) and 2.07 ng/mL (Area Under the Curve 0.860), respectively. Briefly, serum Tg and Cyfra 21.1 levels above these cut-off levels were found in 61 and 46 of 62 sREC cases and 76 and 4 of 376 NED cases, respectively. Sensitivity, specificity, accuracy, positive (PPV) and negative (NPV) predictive values were 98%, 80%, 82%, 44%, 100% for Tg and 74%, 99%, 98%, 92%, 96% for Cyfra 21.1. Positive Tg and Cyfra 21.1 values were found in 36/37 (97%) and 22/37 (59%%) patients with loco-regional recurrences and 25/25 (100%) and 24/25 (96%) patients with distant metastases, respectively (Chi-square test: Tg *ns*, Cyfra 21.1 p < 0.0001). Both markers were significantly increased in sREC [Tg: 3.37 (0.32–3420) ng/mL; Cyfra 21.1: 2.81 (0.65–275 ng/mL)] compared to NED patients [Tg: 0.15 (0.1–92.2) ng/mL; Cyfra 21.1: 1.12 (0.30–2.56) ng/mL (Tg p < 0.0001; Cyfra 21.1 p 0.0004) and in patients with distant metastases [Tg: 11.15 (0.60–3420) ng/mL; Cyfra 21.1: 3.34 (0.65–275) ng/mL)] compared to those with loco-regional recurrences [Tg: 4.49 (0.32–28.2) ng/mL; Cyfra 21.1: 1.29 (0.71–7.35) ng/mL] (Tg p < 0.0001; Cyfra 21.1 p 0.0004), respectively (Fig. 1). As shown in Fig. 2, Tg levels <0.35 ng/mL and Cyfra 21.1 levels <2.07 ng/mL were associated to significantly longer disease-free survival while higher values were significantly associated to structural recurrences and disease-related deaths over time [Odds’ Ratios: Tg 325.27 (95%CI 19.82–5337.39), p > 0.0001; Cyfra 21.1 300.84 (95%CI 90.51–999.89) p < 0.0001]. The impact of combined Tg and Cyfra 21.1 results is showed in Fig. 3. Finally, no significant relationship was found between the two markers.

### Multivariate analysis

The multivariate analysis included serum Tg and Cyfra 21.1 levels and the other clinico-pathologic parameters significantly associated with recurrence; i.e. pT, pM, age > 55 yrs, and high risk ATA category (Table 3). The model showed significant results (p < 0.001) with serum Tg and Cyfra 21.1 as the most accurate independent predictors of tumor relapse (p < 0.0001). Out of the other features pT, TNM stage, and age > 55 yr retained levels of significance higher than that the specific multivariate model, while pM1 and high risk classification were not correlated with disease recurrence.

**Table 3:** Multivariate analysis (significance of model for p < 0.001).

	Coefficient	Standard Error	t	p
TNM stage	−0.02084	0.007211	−2.890	0.0040
Age > 55 years	0.03346	0.01533	2.183	0.0295
Tg > 0.35 ng/mL	0.14350	0.01823	7.868	<0.0001
Cyfra 21.1 > 2.07 ng/mL	0.76020	0.03149	24.142	<0.0001

## Discussion

It was well demonstrated that DTC cancer cells express cytokeratin-19^8, 9^ which is one of the various kinds of cytokeratins comprising the intermediate filaments of the cytoskeleton^22^. Cyfra 21.1 is a fragment of cytokeratin-19 that is likely released into the extracellular space during the intermediate stage of epithelial cell apoptosis^23^ and can be detected in serum by using anti-Cyfra 21.1 antibody^24^. Previous reports postulated a role for circulating cytokeratins fragments in predicting more aggressive DTC biology. In our series neither distant metastases nor disease-related deaths occurred in patients having serum Tg levels <0.35 ng/mL and only one false-negative Tg result was observed in a patients having a local recurrence. On the other hand, negative Cyfra 21.1 levels were found in all patients with NED at final follow-up but serum Tg > 0.35 ng/mL. Finally, high levels of serum Cyfra 21.1 were observed in 59% of those patients with loco-regional recurrences and 96% of those harboring distant metastases. Accordingly, the overall diagnostic accuracy increased by measuring Cyfra 21.1 in patients with serum Tg levels >0.35 ng/mL and high levels of Cyfra 21.1 confer an increased risk of distant spreading of the disease. This is likely due to a progressive development of more aggressive phenotype as the number of chromosomal abnormalities increases and disease-specific survival decreases in patients harboring distant DTC metastases compared to primary tumours and loco-regional recurrences^25–28^. On these basis, as the main result of the present study, Cyfra 21.1 levels exceeding 2.07 ng/mL significantly increased the risk of disease related recurrence and death over time while lower leves are associated to favorable outcome even in the presence of incomplete Tg response at early follow-up.

Strengths of our study are the large number of patients, the homogeneous treatment and follow-up protocols with uniform diagnostic methods, risk-assessment and dynamic risk stratification criteria. Some potential limitations should be also addressed; first, while consecutive patients were enrolled and prospectively managed, the performance of Cyfra 21.1 measurement was evaluated by *post-hoc* analysis. However, Cyfra 21.1 levels were not taken into account in clinical management and relevant biases are unlikely. Also, the period of postoperative follow-up of our patients might not be considered as long enough; nevertheless, most relapses occur early during follow-up and recurrences have previously been reported to be rare in patients achieving excellent response after thyroid ablation. Then, no significant bias might be expected. Our study was not designed to test the diagnostic performance of Cyfra 21.1 in patients carrying positive TgAb. In these cases Tg is considered not reliable and TgAb disappearance kinetics after treatment are widely adopted as surrogate tumour marker^29^. Then, future comparisons of Cyfra 21.1 and TgAb kinetic in TgAb-positive DTC patients appear desirable.

## Conclusions

In conclusion, our study proved that Cyfra 21.1 is a tool available to independently predict survival of DTC patients not achieving excellent response after primary treatment.

## Methods

### Patients, samples, study design and reference standard

The medical records of all DTC patients diagnosed, treated and followed-up since January 2005 in participating centers were reviewed to extract a standardized dataset (i.e, demographic data, surgical and pathological report, follow-up reports including imaging and laboratory data). Then patients were enrolled for the present study if: 1. they underwent a (near-)total thyroidectomy (and, on indication, central neck lymph node dissection) with a histologically confirmed DTC; 2. serum Tg and Tg autoantibodies (TgAb) measurements were obtained 6 to 12 months after ablation and 3. residual serum samples >0.5 mL were available; 4. serum samples were stored at −80 °C and undergone less than 1 freeze/thaw cycle, 5. coexisting diseases potentially causing falsely positive Cyfra 21.1 measurements were excluded [i.e. any type of lung cancer and mesothelioma, breast cancer, bladder cancer and squamous cell cancers; liver diseases;chronic kidney diseases and cutaneous systemic diseases (i.e. pemphigus, psoriasis). Follow-up visits consisted in a clinical examination with neck US and basal serum Tg determination on a yearly basis. Additional examinations were performed on indication in selected cases. A disease status was assigned by attending physicians for each follow-up visit basing on the longitudinal review of the available clinical, imaging, biochemical and cytological/histological data. Patients were classified as alive with no evidence of disease (NED) if there was no clinical, imaging, or cytological/histological evidence of disease and their measured basal Tg levels were undetectable (i.e. below the functional sensitivity of the locally employed assay) or, if detectable, they were less than 1 ng/mL and decreased or remained unchanged over time^30^. Patients who did not fulfill these criteria were classified as alive with disease (i.e. recurrence, REC) and further stratified into alive with incomplete biochemical response (bREC) or alive with structural recurrence (sREC), respectively. The overall survival (OS) was calculated from the date of radioiodine ablation to the date of disease-related death. The disease-free survival (DFS) was calculated from the date of radioiodine ablation to the date of last follow-up (NED patients) or the date of relapse detection (bREC and sREC patients), respectively.

### Laboratory

Once the study was completed frozen sera aliquots were centralized at Department of Clinical Chemistry and Laboratory Medicine, Ente Ospedaliero Cantonale, Bellinzona (Switzerland). Serum Tg, Tg autoantibodies (TgAb) and Cyfra 21.1 were measured by specific Cobas® assays on fully automated Elecsys® platform (Roche Diagnostics, Penzberg, Germany). The Tg assay is standardized 1:1 against BCR© 457 international standard (BCR, Brussels, Belgium) and display a functional sensitivity of 0.1 ng/mL; the TgAb assay is standardized against the WHO 65/93 international standard and display a functional sensitivity of 20 IU/mL; the Cyfra 21.1 assay displayed a limit of detection (LoD) of 0.10 ng/mL as reported by the manufacturer in assays’ insert packages. Given kits were used to measure all samples and measurements were done strictly following manufacturer’s instructions.

### Statistical analysis

Mann-Whitney U, Wilcoxon test, and paired or unpaired t-test were used to analyze differences between paired or unpaired variables in two groups of patients. The predictive tests, i.e. sensitivity, specificity, positive (PPV) and negative (NPV) predictive value, and accuracy, were calculated according to Galen and Gambino. Agreement between continuous variables was assessed by Passing and Bablok regression analysis. Continuous variables were dichotomized by receiver operating characteristics (ROC) curve analysis using the maximum value of Youden’s index (J) as the most accurate cut-off point. Aging was analyzed in several fashions: as a continuous variable (comparison of medians) or by using the specific cut-offs of 45 years or 55 years (Links TP *et al*.^28^). DFS was estimated by using the Kaplan-Meier method and differences between curves were analyzed by log-rank or Mantel-Haenszel test and expressed as Hazard Radio (HR).The association degree of specific parameters with cancer relapse was assessed by Odds Ratio (OR). Parameters with significant association with cancer recurrence were included to carry out a model for a multivariate regression analysis. Statistical significance was set at p < 0.05. All statistical tests were performed by MedCalc Statistical Software, version 15.8 (MedCalc software bvba, Ostend; Belgium).

### Ethics

The protocol was approved by the Ethics Committee of Canton Ticino (Switzerland) and Clinical Research Committees of Ente Ospedaliero Cantonale (EOC), Bellinzona (Switzerland). All patients gave their informed consent before participating in the study. All experiments were performed in accordance with relevant guidelines and regulations.

### Data availability

The datasets generated during and/or analyzed during the current study are available from the corresponding author on reasonable request.
